# Improved USER cloning for TALE assembly and its application to base editing

**DOI:** 10.1371/journal.pone.0289509

**Published:** 2023-08-04

**Authors:** Jizeng Zhou, Jiaowei Wang, Fangbing Chen, Zhenpeng Zhuang, Min Chen, Yang Yang, Xian Luo, Chengcheng Tang, Xiaoqing Zhou, Yue Chi, Jinling Wang, Yu He, Kun Zhang, Qingjian Zou

**Affiliations:** 1 School of Biomedical and Pharmaceutical Sciences, Guangdong University of Technology, Guangzhou, China; 2 CAS Key Laboratory of Regenerative Biology, Guangzhou Institutes of Biomedicine and Health, Chinese Academy of Sciences, Guangzhou, China; 3 Guangdong Provincial Key Laboratory of Large Animal models for Biomedicine, South China Institute of Large Animal Models for Biomedicine, School of Biotechnology and Health Sciences, Wuyi University, Jiangmen, China; 4 National Drug Clinical Trial Institution, Jiangmen Central Hospital, Jiangmen, Guangdong, China; University of South Florida, UNITED STATES

## Abstract

Transcription activator-like effectors (TALEs) have been widely used for genome editing, transcriptional regulation, and locus-specific DNA imaging. However, TALEs are difficult to handle in routine laboratories because of their complexity and the considerable time consumed in TALE construction. Here, we described a simple and rapid TALE assembly method based on uracil-specific excision reagent (USER) cloning. Polymerase chain reaction was amplified with TALE trimer templates and deoxyuridine-containing primers. The products were treated with USER at 37°C for 30 min, followed by the treatment of T4 DNA Ligase at 16°C for 30 min. The TALE trimer unit could be rejoined hierarchically to form complete TALE expression vectors with high efficiency. This method was adopted to construct TALE-deaminases, which were used in combination with Cas9 nickases to generate efficient C-to-T or A-to-G base editing while eliminating predictable DNA off-target effects. This improved USER assembly is a simple, rapid, and laboratory-friendly TALE construction technique that will be valuable for DNA targeting.

## Introduction

The transcription activator-like effector (TALE) and the clustered regularly interspaced short palindromic repeat (CRISPR)/Cas9 architecture have been used extensively for mastering and programming gene functions and applied to the advancement of agriculture and medicine. The DNA-binding domain of TALEs comprises an extended array of 34 amino acid (AA) repeats. Each repeat has one of four distinct repeat variable di-residues (RVDs) that differ only at AA positions 12 and 13 and confer nucleotide-binding specificity (NI to A, NG to T, HD to C, and NN or NH to G) [1, 2].

Although CRISPR/Cas9 is currently the most used system for gene editing due to its simplicity and convenience [3, 4], TALE exhibits its own unique advantages for gene targeting. It can be fused not only with the endonuclease FokI, transcriptional regulatory domains [5], and histone modifiers [6], but also with endonucleases and deaminases to edit the mitochondrial genome [7–10]. Therefore, TALEs can be used in the clinical treatment of mitochondrial diseases. Furthermore, the recognition range of TALE nucleases (TALENs) is from 10 to 31 (×2) base pairs (bp), and off-target risks are nearly lacking [11, 12]; thus, TALEs are appropriate for clinical applications. However, popular protocols for TALE construction are based on Golden Gate cloning [13], solid-phase assembly [14–16], and Gibson assembly [17, 18], which are either time-consuming or technically difficult. This issue widely hinders the use of TALEs in gene modification.

We recently generated TaC9-BE, the base editor with dual guides, including Cas9/sgRNA and TALE, which induced efficient C-to-T or A-to-G conversion without detectable predictable DNA off-target mutations [19, 20], providing a safe tool for gene therapy and the generation of genetically modified organisms. However, the complexity of TALE construction is the primary obstacle to TaC9-BE application.

Here, we improved uracil-specific excision reagent (USER)-based cloning for TALE assembly. USER is composed of uracil DNA glycosylase, which catalyzes the excision of a uracil base and forms an apyrimidinic (AP) site, while endonuclease VIII breaks the phosphodiester backbone at the 3ʹ and 5ʹ sides of the AP site to release base-free deoxyribose. TALE trimers [18] were amplified via polymerase chain reaction (PCR) by using deoxyuridine (dU)-modified primers and assembled in order through improved USER (iUSER) cloning. This method has achieved rapid and efficient TALE assembly without the limitation posed by the Type II restriction enzyme in Golden Gate cloning. The constructed TALE vectors rapidly accelerated the TaC9-BE system upon the application of base editing.

## Materials and methods

### Vector construction

TALE backbone with enhanced green fluorescent protein (EGFP) and the TALE Trimer Library were obtained from our previous research [18]. All USER primers were synthesized by Guangzhou IGE Biotechnology Ltd. and amplified with Q5U^®^ Hot Start High-Fidelity DNA Polymerase (New England Biolabs, #M0515). All PCR products with dU were excised with Thermolabile USER® II Enzyme (New England Biolabs, #M5505) and assembled with T4 DNA Ligase (New England Biolabs, #M0202) or Hi-T4 DNA Ligase (New England Biolabs, #M2622). In constructing all the single-guide RNA (sgRNA), two complementary oligonucleotides were synthesized by Guangzhou IGE Biotechnology Ltd., annealed via PCR (Bio-Rad T100 thermal cycler), and cloned into the BbsI site of the sgRNA expression vector by using T4 DNA Ligase (New England Biolabs, #M0202). All sgRNA and TALE target sequences are listed in S3 Table. Fully assembled plasmids were generated during the reaction and then directly transformed into 25 μL of chemically competent bacteria and plated for overnight growth. Bacterial colonies were collected and cultured for another 8 h in liquid lysogeny broth medium. Plasmids were extracted by using QIAprep Spin Miniprep Kit (Qiagen). The assembled plasmids were identified via PCR and electrophoresis. Full-length products were sequence-verified through Sanger sequencing.

pCMV-ABE8E^DelR153^-mcherry was constructed on the basis of pCMV-YE1-BE4max-mcherry which was obtained from our previous research. All subcloning experiments were conducted with DH5α competent cells. All restriction endonucleases were purchased from Thermo Fisher Scientific.

### Cell culture and transfection

Human HEK293T and Rabbit fibroblasts cells were cultured in Dulbecco’s modified Eagle’s medium (HyClone) supplemented with 10% fetal bovine serum (Gibco) at 37°C under 5% CO_2_. Then, they were seeded on 24-well plates (Greiner Bio-One) and transfected at approximately 90% density. After 24 h, 250 ng of sgRNA, 500 ng of plasmids with nCas9, and 300 ng of plasmids with TALE were transfected using 1 μl of Lipo8000 Transfection Reagent (Beyotime, C0533) per well. After 3 days of transfection, cells were collected and filtered through a 40 μm strainer (BD Falcon), and the transfected cells were collected on a Beckman Coulter MoFlo Astrios Cell Sorter (laser option: blue 488 nm and red 561 nm). About 10 μl of NP40 Lysis Buffer was added to the collected cells, and the reaction was incubated at 56°C for 1 h. The products were used as a template for amplification.

### Targeted amplicon sequencing and analysis

The target sequence was amplified from the cell lysate by PCR (2× PhantaMax Master mix, Vazyme) with specific primers. The products were then sent out for Sanger sequencing (Guangzhou IGE Biotechnology Ltd.). The PCR results of amplicon were quantified using EditR. All PCR primers are listed in S1–S3 Tables.

### Statistical analysis

The GraphPad Prism software (version 8.4.3) was used for data analysis. The data are presented as the mean ± SEM. All tests conducted were two-way ANOVA-test, and the difference was considered significant at *p < 0.01.

## Results

### Rapid assembly of TALE trimers with iUSER cloning

**Fig 1:**
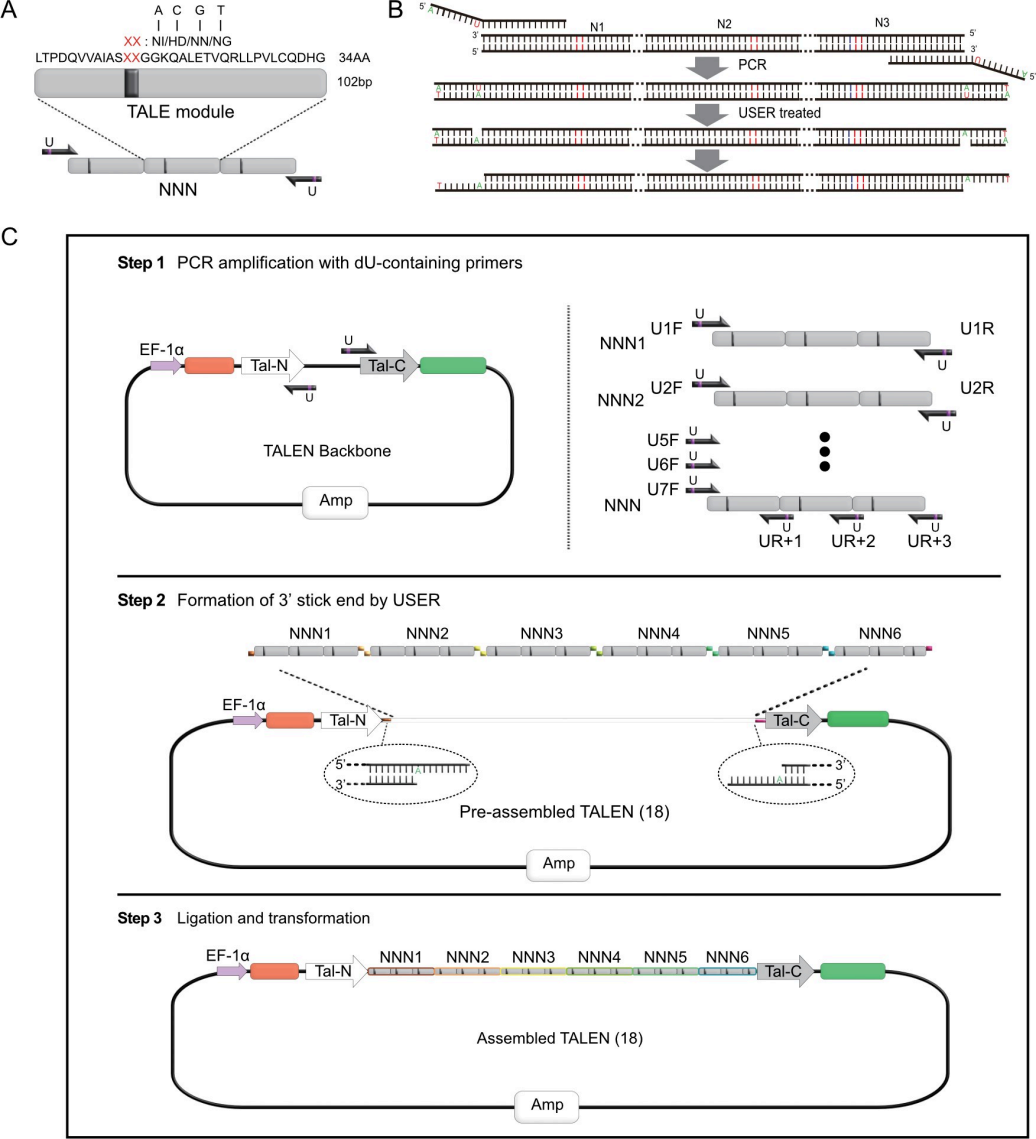
Schematic of TALE construction by iUSER cloning. A) TALE trimer with three repeats. Each repeat contained 34 AAs with one of the four different RVDs. Correspondingly, the coding DNA sequence contained 102 bp. B) Schematic of PCR and iUSER treatment to generate 3’ overhangs. C) Strategy for three steps of TALE vector assembly by iUSER cloning.

A TALE repeat contains 34 AAs with RVD at positions 12 and 13 (NI, NG, NN, and HD). We developed a TALE trimer library that contained 64 combinations for targeting NNNs (N: A, C, G, or T; Fig 1A) [18]. Degenerated codons were used to reduce the number of repetitive sequences and lower the rock for TALE construction. Here, iUSER, an organic combination of trimers and USER cloning, was applied to TALE assembly. Trimers were used as templates, while special primers were synthesized with dU insertion at the eighth to tenth nucleosides that accede the site of T in the templates from the 5ʹ end. The last nucleoside at 5ʹ end was A. PCR products were treated with USER enzyme to generate a single nucleotide gap at the location of dU. Finally, 3ʹ overhangs that contained eight to ten oligonucleotides were formed (Fig 1B).

The construction process of TALE vectors is simple and fast when iUSER cloning is adopted. First, TALE backbone and trimers were amplified with dU-containing primers. Six ordered primer pairs (OPPs), namely, U1-F/R, U2-F/R, U3-F/R, U4-F/R, U5-F/R, and U6-F/R, were generated, each with an 8–10 bp overlap at their 5ʹ ends before dU (S1 Table). The first primer 1F shared an overlap sequence with the front end of the backbone TALE vectors. The three last unique reverse primers, i.e., UR+1, UR+2, and UR+3, were designed to target the first, second, and third monomers of the last trimer module, respectively. The 5ʹ ends of these primers overlapped with the other end of the backbone vectors (Fig 1C, top row). Trimers from the library were chosen in accordance with the TALE target sequences and used for PCR with the OPPs. Second, the PCR fragments of the backbone vectors and trimers were ligated in order through USER cloning and ligation. (Fig 1C, middle and bottom rows). USER created a single nucleotide gap at each dU location, resulting in PCR fragments flanked with single-stranded extensions and allowing for the seamless and orderly assembly of the TALE trimer into vectors. TALE assembly is accomplished after only 2 days.

**Fig 2:**
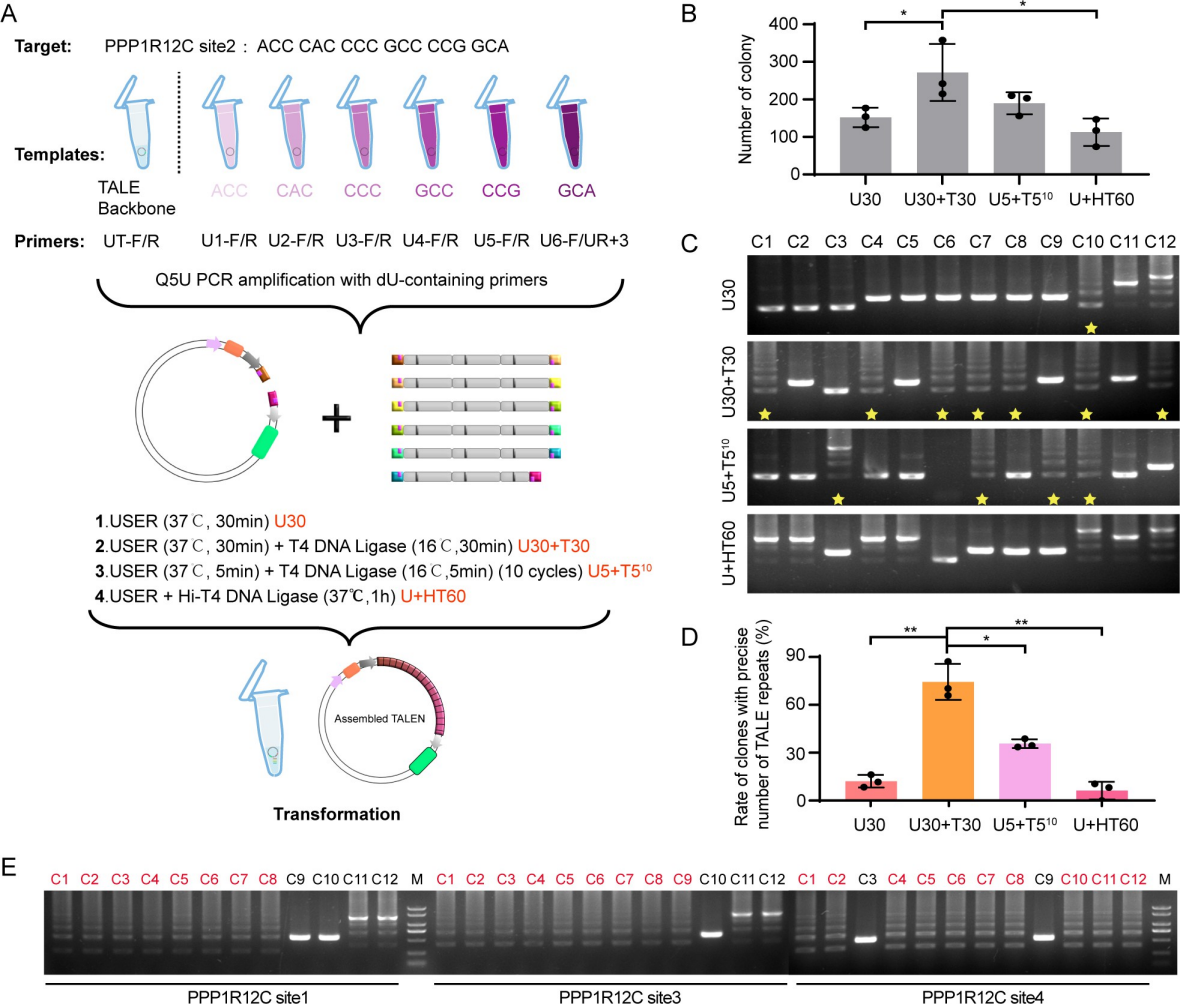
Construction of TALE vectors with different conditions. A) Summary of the TALE vector assembly by iUSER cloning. Four different conditions were listed. B) The number of bacterial colonies on agar plates constructed using the four different TALE vector schemes. C) Electrophoresis of PCR fragments from 4 different groups. The clones highlighted in yellow star represent the correct PCR products which formed a list of DNA ladder bands (~300bp interval) on gel electrophoresis. C1–C12, the identified colonies. D) The statistics of precise TALE assembly rates in four groups. E) Electrophoresis of PCR fragments with optimized condition. The clones highlighted in red colors represent the correct PCR products which formed a list of DNA ladder bands. M, Markers. For B and D, data are shown as individual data points and mean ± SEM for n = 3 different transformation experiments performed on different days. *p < 0.05; **p<0.01.

The common TALE length is about 12–21 repeats. Here, the construction of 18 TALE repeats for targeting Protein Phosphatase 1 Regulatory Subunit 12C (PPP1R12C) site 2 (ACCCACCCCGCCCCGGCA) was presented as an example. The trimers of ACC, CAC, CCC, GCC, CCG, and GCA were amplified using dU primers U1-F/R, U2-F/R, U3-F/R, U4-F/R, U5-F/R, and U6-F/UR+3, respectively. The TALE vector was amplified with primer UT-F/R. The linearized backbone and six ordered trimers with dU were treated with USER and mixed for ligation. Four schemes were designed as follows (Fig 2A): Group 1 (USER was treated at 37°C for 30 min, U30), Group 2 (USER was treated at 37°C for 30 min, followed by the treatment of T4 DNA Ligase at 16°C for 30 min, U30+T30), Group 3 (USER and T4 DNA Ligase were mixed and treated for 10 cycles at 37°C for 5 min and 16°C for 5 min, U5+T5^10^), and Group 4 (USER and Hi-T4 DNA Ligase were mixed and treated at 37°C for 1 h, U+HT60).

Bacterial transformation was performed to generate correct and abundant TALE vectors. Groups 2 and 3 had more colonies than the other groups (Fig 2B and S1 Fig). Subsequently, 12 colonies were selected from each group for PCR identification. The PCR products with correct templates formed a list of DNA ladder bands (~300 bp interval) on gel electrophoresis due to the six trimer repeats. We determined that 7 of the 12 products in Group 2 had 6 bands, 3 of the 12 products in Group 3 also had 6 bands, and no corrected band was found in the other 2 groups (Fig 2C). More colonies were analyzed via PCR, and the statistical results also showed that Group 2 had the highest corrected bands (>60%), followed by Group 3 (~30%, Fig 2D). Each TALE vector with corrected bands was verified through further sequencing. On the basis of statistical data, the conduction of Group 2 was used in subsequent experiments. To verify the reproducibility of USER cloning for TALE vector construction, we designed 3 more TALE vectors with 14, 15, and 17 repeats (targeting PPP1R12C sites 1, 3, and 4, respectively). Gel electrophoresis indicated that the precision rate of each TALE construction was more than 66% (Fig 2E).

### TALEs generated by iUSER cloning are applied to base editing

**Fig 3:**
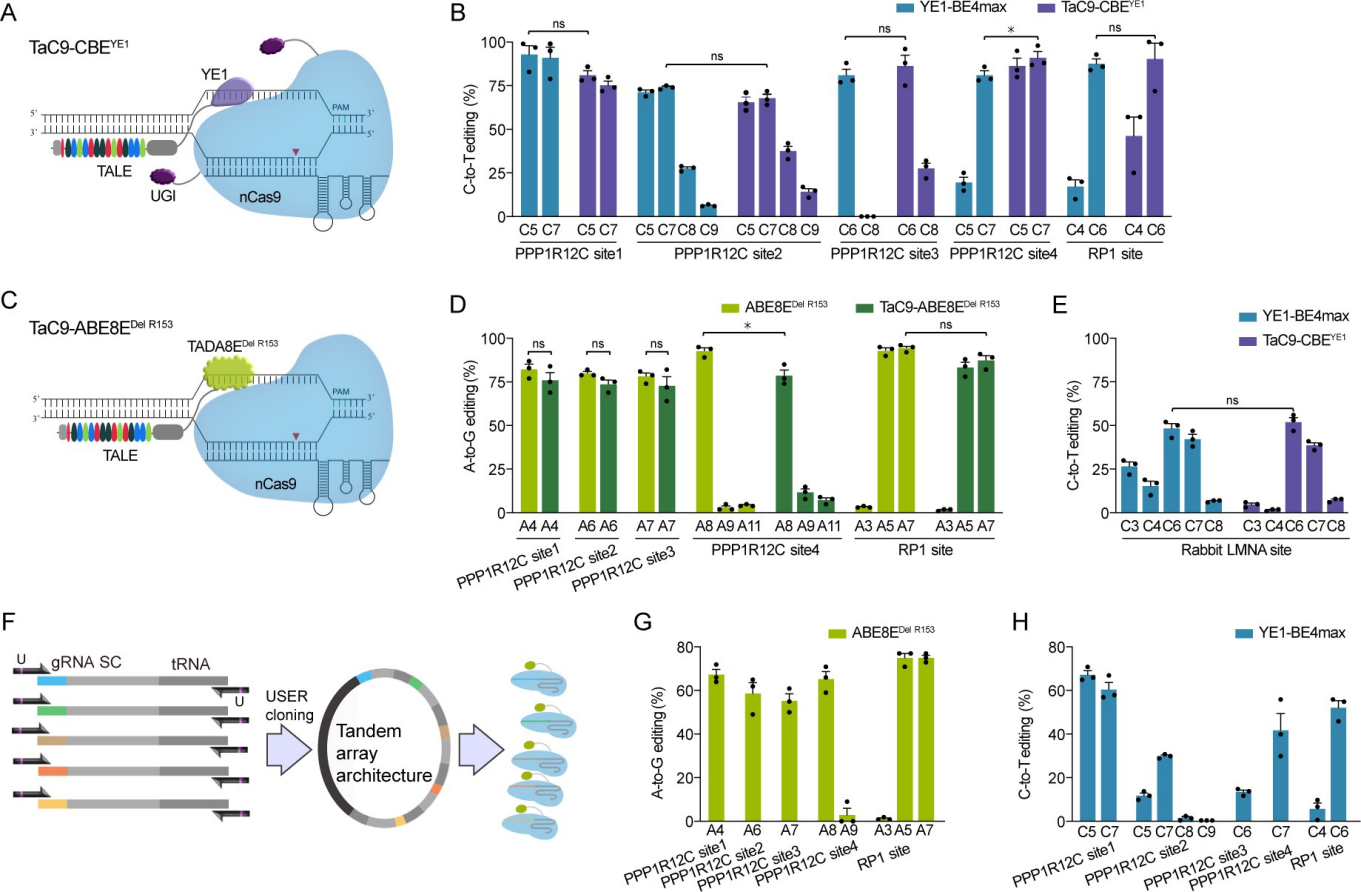
Efficient base editing using the TALE and tandem sgRNA constructed by iUSER cloning. A) Schematic of target effects TaC9-CBE^YE1^. B) On-target editing efficiency of YE1-BE4max and TaC9-CBE^YE1^ across five genomic loci in HEK293 cells. C) Schematic of target effects TaC9-ABE8E^DelR153^. D) On-target editing efficiency of ABE8E^DelR153^ and TaC9-ABE8E^DelR153^ across five genomic loci in HEK293 cells. E) On-target editing efficiency of YE1-BE4max and TaC9-CBE^YE1^ on rabbit LMNA gene locus. F) Schematic of tandem sgRNAs construction using iUSER cloning. G) On-target editing efficiency of ABE8E^DelR153^ across five genomic loci in HEK293 cells. H) On-target editing efficiency of YE1-BE4max across five genomic loci in HEK293 cells. For B, D, E and G, data are shown as individual data points and mean ± SEM for n = 3 different transfection experiments performed on different days. *p < 0.05 by two-way ANOVA, ns = no significance.

To test the functionality of the TALE vectors assembled via iUSER cloning, the four TALEs constructed above and a new TALE that targeted the Oxygen-regulated protein 1 (RP1) locus were tested with nCas9/guide RNA (gRNA) in the TaC9 system. The target site sequences are listed in S2 Table. First, the five TALEs were separately fused with the cytidine deaminase variant YE1. TaC9-CBE^YE1^ consisted of TALE-YE1s and nCas9/gRNA, and both guides were designed to target the same gene loci (Fig 3A). HEK293T cells were transfected with TaC9-CBE^YE1^ or YE1-BE4max and collected for sequencing. TaC9-CBE^YE1^ and YE1-BE4max had high C-to-T edit efficiency (>70%) in all five target sites (Fig 3B). Similarly, TALEs were fused with the adenosine deaminase variant ABE8E^DelR153^ and combined with nCas9/gRNA to form the TaC9-ABE8E^DelR153^ system (Fig 3C). TaC9-ABE8E^DelR153^ was transfected into HEK293T cells, and the sequencing showed that A-to-G edit efficiency reached up to 75% in all five target sites, similar to that of the single-guide editor ABE8E^DelR153^ (Fig 3D). To further validate the universality of the iUSER-constructed system on different types of cells, we tested the base editing efficiency of Lamin A/C (LMNA) locus in rabbit fibroblast cells. The editing window and efficiency of TaC9-CBE^YE1^ were comparable with those of YE1-BE4max (Fig 3E). The results indicated that the TALEs assembled via iUSER cloning are not only simple and efficient, but also exhibit functional activity for target DNA binding.

### Multiple gRNAs generated by iUSER cloning are applied to base editing

In addition to TALE construction, USER cloning was used to produce five sgRNA (targeting human endogenous PPP1R12C sites 1 to 4 and RP1 locus) from a single polycistronic gene. The transfer RNA (tRNA) was engineered to separate each sgRNA [21]. Then, tRNA–gRNA units were amplified with dU-containing primers (S3 Table) and ligated in tandem with USER cloning to form tandem sgRNA expression. Finally, tRNA–gRNA arrayed architecture and Cas9-deaminases were co-transfected with cells to express five sgRNA and Cas9-deaminases simultaneously (Fig 3F). For A-to-G base editing, the tRNA–gRNA architecture was co-delivered with ABE8E^DelR153^, resulting in high-efficiency A-to-G conversion (60%) in all five sites (Fig 3G). For C-to-T base editing, the tRNA–gRNA architecture was co-delivered with YE1-BE4max. Sequencing showed that C-to-T conversion efficiency was more than 40% in three out of five sites and between 15% and 40% in two other sites (Fig 3H). This result indicates that USER cloning is not only applied to TALE construction but also to multiple gRNA construction.

## Discussion

In this study, we used iUSER cloning to construct either TALE or gRNA tandem vectors with a simple, rapid, and efficient process. Although USER cloning has been used in TALE assembly, it can only form pentamers, and thus, another step of Golden Gate cloning is required to finish only half of the whole TALE assembly [22]. The two-step method results in complex and time-consuming TALE assembly. Our single-step iUSER cloning can construct TALEs in a simple and rapid manner based on the existing TALE trimer library.

The USER cloning-rejoined TALE repeats formed pentamers in a ligation-independent manner similar to other ligation-independent cloning [22, 23]. However, we found that T4 DNA Ligase is indispensable for efficient iUSER TALE assembly. The optimized TALE assembly was USER-treated for 30 min followed by ligation for 30 min, distinguishing it from Golden Gate cloning, which has a circulation between two temperatures. Golden Gate cloning needs Type IIS restriction endonucleases to release four base overhangs, which require optimization for cloning, because T4 DNA Ligase tolerates 2 bp mismatches, gaps, and other imperfect structures with varying efficiency levelsy [24]. iUSER cloning created 8 to 10 base overhangs, providing more choices with less mismatches between each fragment. These overhangs are more likely to form Watson–Crick overhang pairs, ensuring high-fidelity TALE assembly.

Both TALE and CRISPR/Cas9 are powerful tools for genome editing. However, TALE technology has some advantages over CRISPR/Cas9 in certain aspects. Firstly, due to gRNA may tolerate to mismatched DNA targets, CRISPR/Cas9 could cause the potential off-targets, that cuts DNA at unintended loci. In contrast, TALE recognizes DNA targets through specific protein-DNA interactions, leading to a lower likelihood of off-target effects [25]. Secondly, Cas9 requires a target-adjacent sequence, the PAM, to bind DNA, which has greatly limited its application on targets lack of PAM [26]. TALE recognizes DNA by one-to-one correspondence of RVDs and nucleotides, can bind any DNA target sites [25]. Thirdly, the large size of CRISPR/Cas9 makes it difficult to deliver into cells using AAV vectors [27]. TALE is smaller and can change size according to actual needs, which makes it easier for delivery. Lastly, the all-protein structure of TALE offers the advantage of both nuclear DNA and mitochondrial DNA editing [7–10].

In summary, TALE technology has advantages over CRISPR/Cas9 in terms of target specificity, lower risk of off-target effects, and smaller size. Here, we developed a novel method to construct TALE with simple and rapid way, which would assist TALE for wildly using on gene targeting.

## Supporting information

S1 Fig Bacterial colonies grown on agar plates were constructed using four different TALE vector schemes. (DOCX)

S2 Fig Representative sequencing results of five human gene sites and one rabbit gene site edited using ABEs or CBEs. (DOCX)

S1 Table Primers used for TALE assembly and identification. (DOCX)

S2 Table The sequences dual guider gRNA and TALE target sites. (DOCX)

S3 Table Primers used for gRNA tandem assembly and identification. (DOCX)

S1 Raw images (PDF)
