# Supplementary material for: Improved USER cloning for TALE assembly and its application to base editing
Source: PLoS One. 2023 Aug 4;18(8):e0289509. doi: 10.1371/journal.pone.0289509 (PMC10403120; doi:10.1371/journal.pone.0289509)
Supplement: S1 Fig — (DOCX) [file pone.0289509.s001.docx]

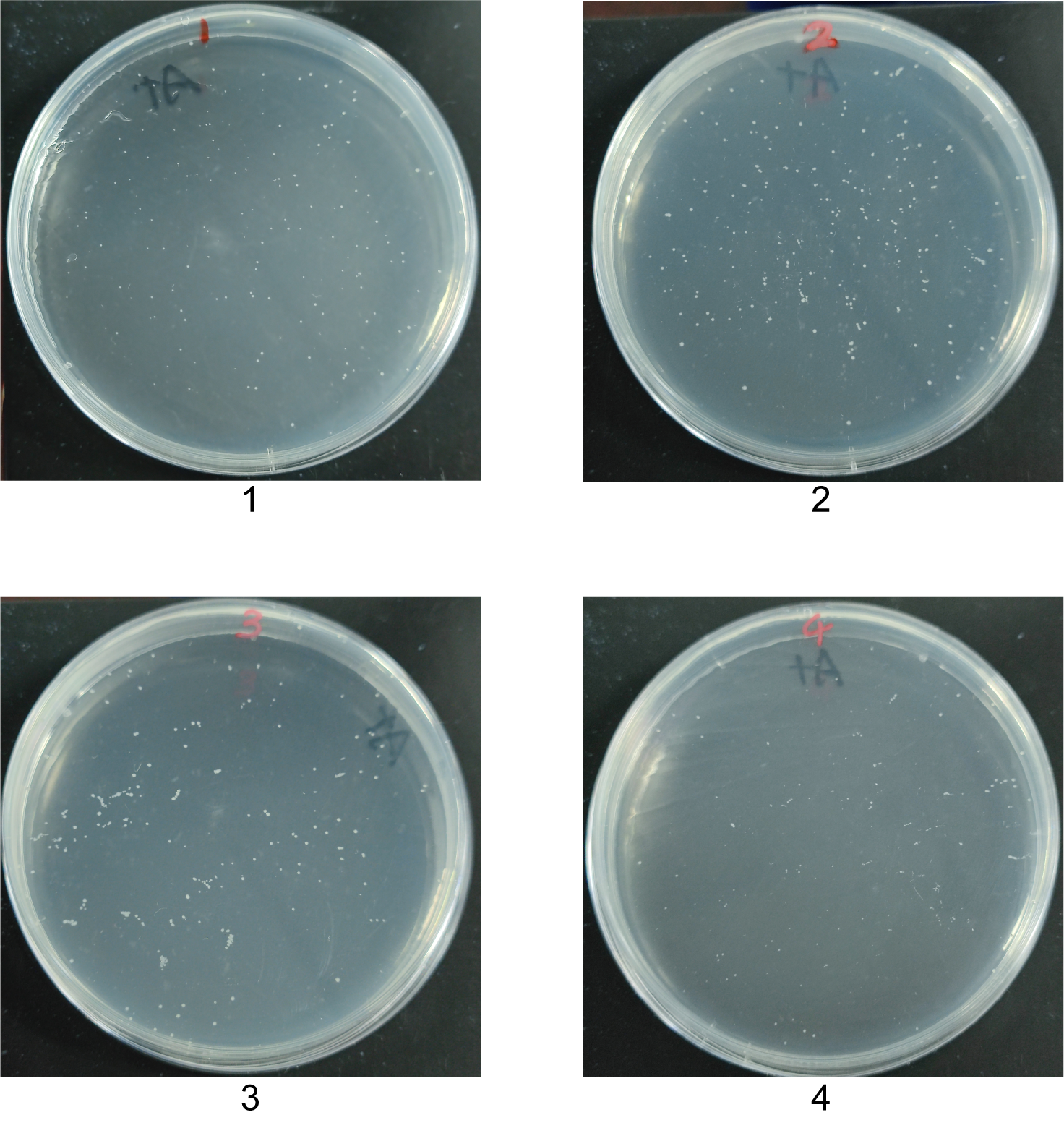


S1 Fig. Bacterial colonies grown on agar plates were constructed using four different TALE vector schemes.

Group 1 (USER was treated at 37℃ for 30 min, U30), Group 2 (USER was treated at 37℃ for 30 min, followed by the treatment of T4 DNA Ligase at 16℃ for 30 min, U30+T30), Group 3 (USER and T4 DNA Ligase were mixed and treated for 10 cycles at 37℃ for 5 min and 16℃ for 5 min, and Group 4 (USER and Hi-T4 DNA Ligase were mixed and treated at 37℃ for 1 h).
