# Supplementary material for: Improved USER cloning for TALE assembly and its application to base editing
Source: PLoS One. 2023 Aug 4;18(8):e0289509. doi: 10.1371/journal.pone.0289509 (PMC10403120; doi:10.1371/journal.pone.0289509)
Supplement: S2 Fig — (DOCX) [file pone.0289509.s002.docx]

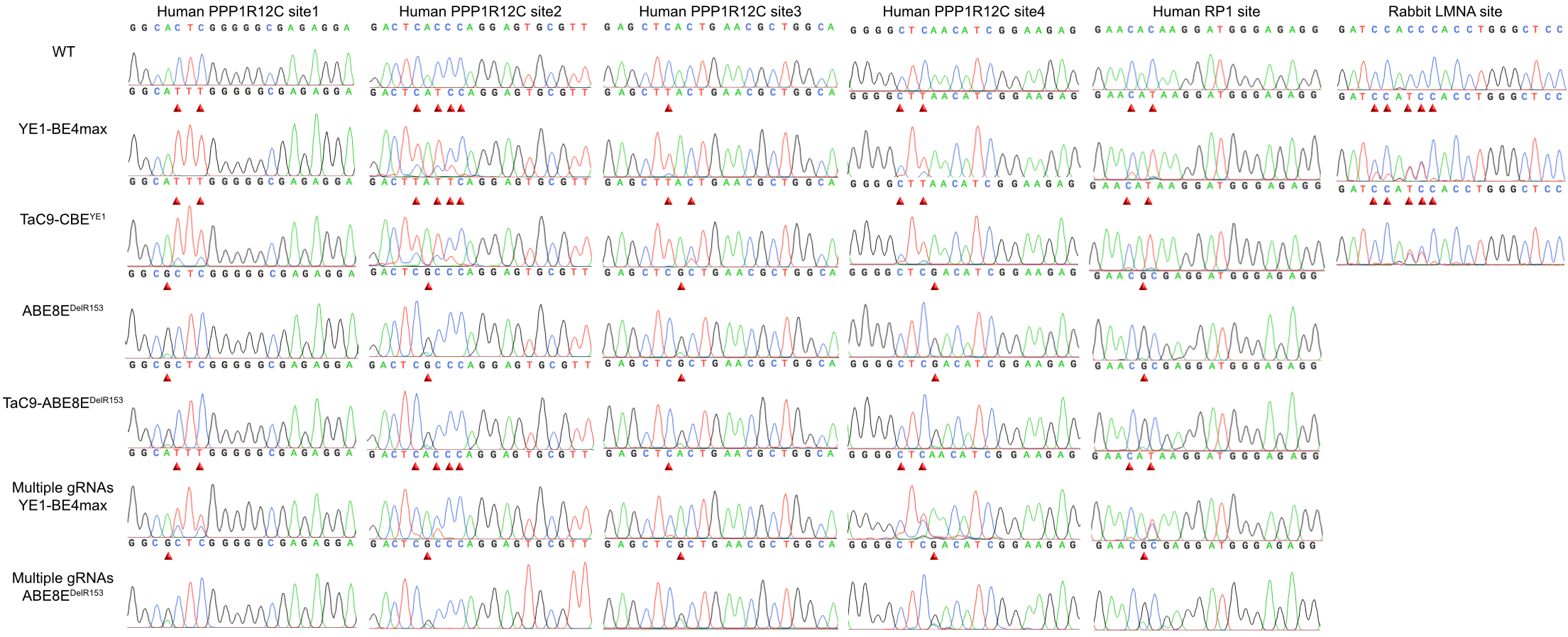


S2 Fig. Representative sequencing results of five human gene sites and one rabbit gene site edited using ABEs or CBEs.

Red triangles highlight the edited bases resulting from ABEs or CBEs.
