# Supplementary material for: Improved USER cloning for TALE assembly and its application to base editing
Source: PLoS One. 2023 Aug 4;18(8):e0289509. doi: 10.1371/journal.pone.0289509 (PMC10403120; doi:10.1371/journal.pone.0289509)
Supplement: S1 Table — (DOCX) [file pone.0289509.s003.docx]

S1 Table. Primers used for TALE assembly and identification.

| Primers | Sequences | Templates |
| --- | --- | --- |
| U1-F | ACTGAACCUGACACCGGATCAAGTTGTC | Trimers |
| U1-R | AGGCCGTGUGCTTGACACAGGACCG | Trimers |
| U2-F | ACACGGCCUGACACCGGATCAAGTTGTC | Trimers |
| U2-R | AACCCATGUGCTTGACACAGGACCG | Trimers |
| U3-F | ACATGGGTUGACACCGGATCAAGTTGTC | Trimers |
| U3-R | AAGGCCGUGTGCTTGACACAGGACCG | Trimers |
| U4-F | ACGGCCTUACACCGGATCAAGTTGTC | Trimers |
| U4-R | AAGACCAUGTGCTTGACACAGGACCG | Trimers |
| U5-F | ATGGTCTUACACCGGATCAAGTTGTC | Trimers |
| U5-R | AGGTGTAAGUCCGTGTGCTTGACACAGGAC | Trimers |
| U6-F | ACTTACACCUGATCAAGTTGTCGCTATTGC | Trimers |
| U6-R | AGGGGTCAAUCCGTGTGCTTGACACAGGAC | Trimers |
| U7-F | ATTGACCCCUGATCAAGTTGTCGCTATTGC | Trimers |
| UR+1-R | ACAATGCUTTCCAATGCCTGCTTCCCAC | Trimers |
| UR+2-R | ACAATGCUTTCGAGCGCTTGCTTGCCGC | Trimers |
| UR+3-R | ACAATGCUCTCCAGGGCTTGTTTTCCGC | Trimers |
| UV-F | AGCATTGUTGCCCAGTTATCTCGCCCTG | TALE backbones |
| UV-R | AGGTTCAGUGGGGCACCCGTCAGTGCA | TALE backbones |
| CEXU-F | AACGTGGCGGCGTGACCGCA | TALE Tandems |
| CEXU-R | GGTGGTCGTTGGTCAACGCGG | TALE Tandems |
