# Supplementary material for: Improved USER cloning for TALE assembly and its application to base editing
Source: PLoS One. 2023 Aug 4;18(8):e0289509. doi: 10.1371/journal.pone.0289509 (PMC10403120; doi:10.1371/journal.pone.0289509)
Supplement: S2 Table — (DOCX) [file pone.0289509.s004.docx]

S2 Table. The sequences dual guider gRNA and TALE target sites.

| Genes | gRNA target sequences | TALE target sequences |
| --- | --- | --- |
| Human PPP1R12C site1 | GGCACTCGGGGGCGAGAGGA | GCCGCCGGAACTCT |
| Human PPP1R12C site2 | GACTCACCCAGGAGTGCGTT | ACCCACCCCGCCCCGGCA |
| Human PPP1R12C site3 | GAGCTCACTGAACGCTGGCA | GACAGGAAGCTCCCA |
| Human PPP1R12C site4 | GGGGCTCAACATCGGAAGAG | GGTCCTGGACTTTGTCT |
| Human RP1 site | GAACACAAGGATGGGAGAGG | GACTTTACTTCCTACTTC |
| Rabbit LMNA site | GATCCACCCACCTGGGCTCC | CTGGCTCTTCCGCCT |
