# Supplementary material for: Improved USER cloning for TALE assembly and its application to base editing
Source: PLoS One. 2023 Aug 4;18(8):e0289509. doi: 10.1371/journal.pone.0289509 (PMC10403120; doi:10.1371/journal.pone.0289509)
Supplement: S3 Table — (DOCX) [file pone.0289509.s005.docx]

S3 Table. Primers used for gRNA tandem assembly and identification.

| Primers | Sequences | Templates |
| --- | --- | --- |
| Ug1-F | ATCCGGUACCAAGGTCGGGCAGGAAG | gRNA-tRNA |
| Ug1-R | ATCCGGUACCAAGGTCGGGCAGGAAG | gRNA-tRNA |
| Ug2-F | AGCTATGCUGGAAGGTCGGGCAGGAAGAG | gRNA-tRNA |
| Ug2-R | ATCGCAGUAGAAAAAAAGCACCGACTCGG | gRNA-tRNA |
| Ug3-F | ACTGCGAUCAAGGTCGGGCAGGAAGAG | gRNA-tRNA |
| Ug3-R | ACGTAGCAUAAAAAAAGCACCGACTCGG | gRNA-tRNA |
| Ug4-F | ATGCTACGUAGAAGGTCGGGCAGGAAGAG | gRNA-tRNA |
| Ug4-R | ACCTTGTCUGAAAAAAAGCACCGACTCGGT | gRNA-tRNA |
| Ug5-F | AGACAAGGUCGGGCAGGAAGAGGGCCT | gRNA-tRNA |
| Ug5-R | AGCTGGGTCUAGAAAAAAAGCACCGACTCG | gRNA-tRNA |
| gRNA-UF | AGACCCAGCUTTCTTGTACAAAGTTGGC | Vector backbone |
| gRNA-UR | ACCGGAUCCAGTCGACTGAATTGGT | Vector backbone |
| gCEXU-F: | GATGGATATCTGCAGAATTC | gRNA Tandem |
| gCEXU-R: | GTAACGGCCGCCAGTGTGCT | gRNA Tandem |
