# Supplementary material for: Improved USER cloning for TALE assembly and its application to base editing
Source: PLoS One. 2023 Aug 4;18(8):e0289509. doi: 10.1371/journal.pone.0289509 (PMC10403120; doi:10.1371/journal.pone.0289509)
Supplement: S1 Raw images — (PDF) [file pone.0289509.s006.pdf]

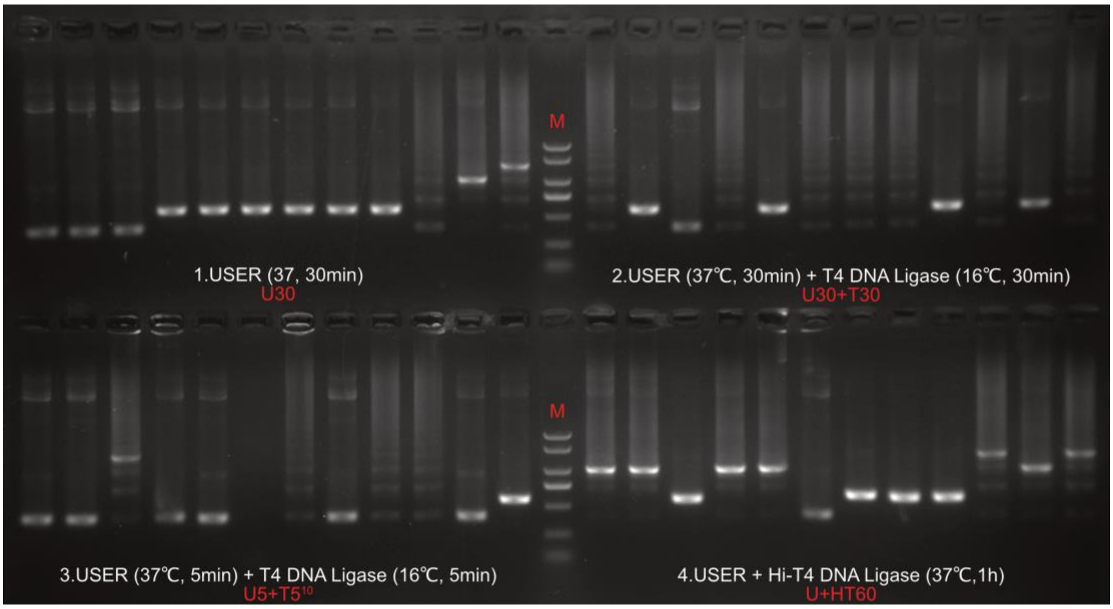


S1_raw_images Fig.1 corresponded to Figure 2C in the manuscript.

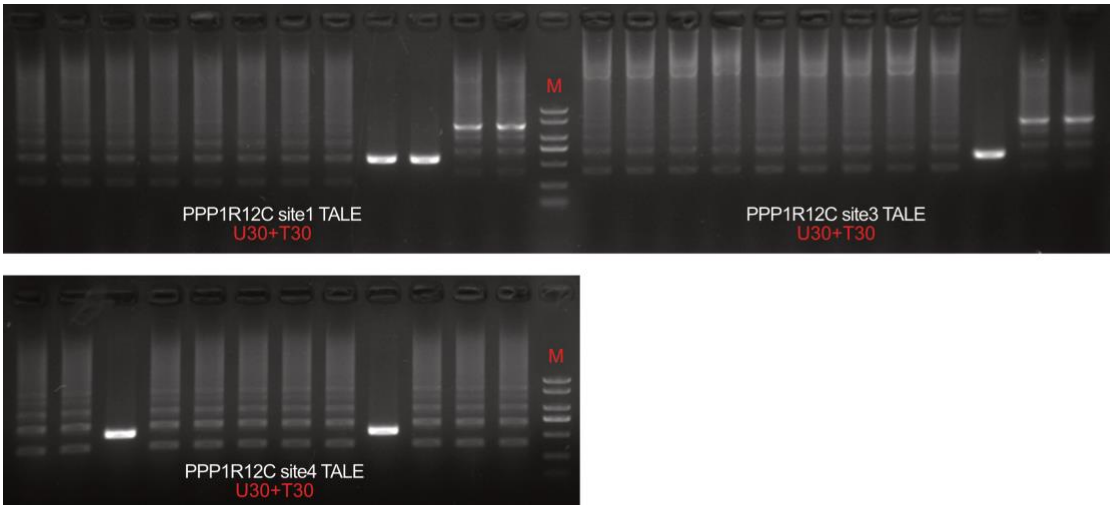


S1_raw_images Fig.2 corresponded to Figure 2E in the manuscript.
